# Digitally connected work and its consequences for strain – a systematic review

**DOI:** 10.1186/s12995-021-00333-z

**Published:** 2021-09-22

**Authors:** Sabrina Zolg, Barbara Heiden, Britta Herbig

**Affiliations:** Institute and Clinic for Occupational, Social and Environmental Medicine, University Hospital, LMU Munich, Ziemsensstr.5, 80336 Munich, Germany

**Keywords:** Digitalization, Work, Strain, Systematic review

## Abstract

**Background:**

Evolving digitization has an impact not only on the organization of work, but also on the health of employees. Dealing with new technologies, integrating new processes and requirements into work, and restructuring tasks among others are demands that can be stressful and impair health.

**Objectives:**

Our aim was to identify (clusters of) working conditions associated with digitally connected work and to analyze their relations with strain, that is, health and well-being outcomes.

**Methods:**

Between May and October 2019, a search string was used to systematically search six databases (EMBASE, Medline, PSYNDEX, PsycInfo, SocIndex, WISO) for German and English texts according to the PEO scheme. The methodological quality was assessed using the Quality Assessment Tool for Studies with Diverse Design.

**Results:**

14 studies were identified. Despite the search string containing latest technologies, we identified mostly studies from the 1980s/90s. To aggregate findings, a categorization of work factors (cognitive demands, social factors, organizational factors, environmental factors) and health factors (motivation/satisfaction, reduced well-being/affective symptoms, physiological parameters/somatic complaints) is introduced. The most frequently identified work factors belong to the category of cognitive demands. For health factors, motivation/satisfaction was identified most often. 475 associations were found in total.

**Conclusions:**

This systematic review provides an overview of work and health factors that have been studied between 1981 and 2019. Recent texts frequently study individualized health factors (e.g., life satisfaction) whereas objective physiological measurement data and objective survey methods such as workplace analysis are not used. This latter approach was predominantly found in the older studies. In order to obtain a comprehensive picture, however, it is worthwhile to use a combination of these subjective and objective approaches for future studies in this field.

## Introduction

The process of automation and digitization has led and still leads to upheavals in the world of work [6, 124]. Primarily prompted by the changes in information processing, ongoing rapid technological advancements maintain efforts to optimize work organization and increase efficiency as well as rationalization (e.g., [24, 103, 123]). With the rise of the internet and wireless networks the use of information and communication technology (ICT) entered a new level as it became possible to work and collaborate – man with man, as well as man with machine – independent of time and place. Currently, algorithm-based self-learning machines become “teammates” [101] with whom it is imperative to deal [49]. These new technological tools actively shape processes, support or replace activities, and enforce or even initiate cooperation [94]. All this has brought about profound changes in the way people work [89].

Work is carried out to an increasing degree in human-machine/human-technology networks which we define in the following as digitally connected work. Such work systems and structures include a large number of players, humans as well as technologies, each following and working in their own logic. They generate a multitude of linkages and interactions as well as various levels of interdependence between these players, and a huge number of simultaneous processes and activities. Thus, work systems like this closely meet definitory criteria of complexity (e.g., [102]). Furthermore, in this kind of digitally connected work, technologies are no longer merely intended to enable and support networking - like in ICT - but also to actively help shape and manage it ([21, 33], see for an overview: [90]). In sum, digitally connected work takes place in human-machine systems which are characterized by digital technologies with inherent logics that can independently and proactively cause interdependencies as well as simultaneities through their interconnectedness with others. However, this can also reduce understandability and manageability of such systems; resulting spontaneous and unexpected „behaviors “can induce a high degree of uncertainty [69, 102].

Numerous studies from work design research (e.g., [11, 25, 63]) show that demands like these can impact strain of employees in the broad sense of short- and longer term somatic, psychological, and behavioral health and well-being (e.g., [50, 67]). Retracing the technical ‘evolution’ of digital connectivity regarding its consequences for health and well-being of employees, three waves can be identified: first, automatization/computerization and the early use of ICT for information processing; second, communication, flexibilization of work in time and place through internet and mobile networks; and third, the development of integration and networking with new, autonomous “teammates” in wide-spread networks.

Research on ICT showed changes in work characteristics over time, e.g., characteristics and increasing amounts of information that need to be processed, an acceleration of work processes, increases in work intensity or in system-related interruptions. They are often accompanied by negative strain reactions of employees, e.g., information overload [20, 54], perceived distress or complaints like fatigue, irritation, and emotional exhaustion [10, 27, 43, 45].

Worktime and workplace flexibility through internet and mobile networks led to numerous research on effects of blurring boundaries between work and private life. In this context, strain is often reported as a result of an ambivalence between work demands and individual needs and requirements. Positive effects resulting from a better integration of work and nonwork domains, such as higher work- and life-satisfaction (e.g., [2, 53, 91]), are reported as well as a negative effects, e.g. impaired recovery and exhaustion [35, 104], distress [12, 41, 81, 113], cognitive and emotional irritation, or other health complaints [14, 60], resulting from work-life-conflict or (expected) permanent availability. Independent from research on blurring boundaries, flexible work schedules constantly showed a negative relation to social support and job satisfaction in data from the European working condition survey, but this same survey also showed that a higher frequency of Internet use is positively associated with employees’ cooperative and self-improvement behavior, as well as job satisfaction [76]. Cooperation between colleagues can profit from the extension of social networks and knowledge transfer [92], but a decrease in face-to-face contact can also reduce social support (e.g., [8]).

The latest developments in the field of autonomous technologies – relevant for digitally connected work focused in this review – put socio-technical systems and their effects back into focus and revive research on technostress. Originally, the term technostress, coined in 1982 by Brod [18], denoted every experience of distress due to ICT-use without any differentiation between stress and strain or consideration of the context. In the work context it later referred to users’ individual characteristics and capabilities to deal with new technologies. A number of so-called “techno-stress” creators (e.g., techno invasion, techno complexity) were introduced and are still used to assess technostress [9, 97, 115]. Dragano et al. [40] and others criticize this as it mixes up different approaches in the consideration of associations between technology and stress. “In some of them, technology is simply an antecedent of other well established work-related stressors like job insecurity or high psychosocial demands whereas in other categories, technology is the primary stressor (e.g., unreliability)” [40]. In view of the increasing variability and complexity of digital and digitally connected systems, we accordingly assume that a more precise focus on work factors and constellations of work factors is necessary for a viable assessment of health risks in such systems, irrespective of the type of activity or attitudes and characteristics of the individual. Additionally, recent reviews on technostress [40, 44, 71, 110] state that most researchers’ view on health outcomes is conditioned by those accessible via self-reporting questionnaires like the one introduced by Tarafdar et al. [115], while other outcomes like physiological parameters are rarely ascertained. Irrespective of the diverse approaches, these reviews conclude that technostress can reduce work and life satisfaction as well as performance and has a negative impact on mental health. As one of the scarce physiological findings, an association of increased cortisol level with high levels of technostress was found [106].

Besides technostress, research on “digitization” (in Germany “Industry 4.0”/ “Arbeit 4.0”) and related effects on employees’ health uses characterizing technologies like cyber physical systems, internet of things, internet of services, augmented manufacturing, robotic etc. [90]. Dependent on the change level, graduations in the complexity of the systems of digitally connected work are found and due to the high number of interrelated socio-technical variables it can be expected that effects on health and well-being might be complex and ambiguous as well (e.g., [116]). In general, research on the impact of these technological changes on health and well-being of employees is still scarce. More often, the transformation is examined considering organizational change or the productivity and effectiveness of work processes (e.g., [24, 103, 123]).

In summary, although a lot of different health related work aspects are known for previous technological developments, a systematic examination of strain-inducing work demands in digitally connected work is missing. As a precondition, work factors and constellations of work factors characteristic for this kind of work need to be identified.

Therefore, the first goal of our systematic review is to provide an overview of (constellations of) working conditions that have been examined in the context of digitally connected working environments so far. The second goal is to consider the influence of these working conditions on the health and well-being of employees. To our knowledge this has not yet been subject of a systematic review. Additionally, the review shall aid to identify the employed occupational health and psychological models as well as research gaps and needs.

## Methods

This systematic review was conducted according to the PRISMA (Preferred Reporting Items for Systematic Reviews and Meta-Analyses) guidelines [83]. It was entered in the International Prospective Register of Systematic Reviews (PROSPERO) under the number CRD42019135431 in July 2019 and last updated in January 2020.

### Information sources

In total, we searched six electronic databases (EMBASE, Medline, PsycInfo, PSYNDEX, SocIndex, and WISO) for eligible published studies. The search ended on 21 October 2019. No studies were added to the data analysis after this date. There were no restrictions on the publication period. Non-original research such as systematic reviews or conference/discussion papers were not taken into account. There were no further eligible studies identified through a manual search. Studies that were not systematically collected, especially grey literature, were not considered in the analysis.

### Search strategy

Our search string was created using the PEO scheme. Population was the working population. Exposure entailed digitally connected work as outlined above. Outcome were strain resp. health effects in general. The operationalization of the search strategy was as follows: we used Mattioli’s [79] search strategy for the working population. To cover the exposure, we chose a broad spectrum of technical terms in the context of digitization and networking. A thematic literature search was carried out to add frequently used terms, for example *cloud computing*, *embedded systems* or *big data*. Terms related to ICT were not included in the search string and studies exclusively dealing with these aspects were excluded as reviews on ICT and health are available (e.g., [13]) and as we consider digitally connected work as a development that goes beyond the pure use of ICT. The search terms *well-being*, *health* and physiolog* for the outcomes were defined to capture all physiological as well as psychosocial health- and well-being-related consequences of strain. The term “technostress” was not included in the search string. As outlined, the concept of technostress but also questionnaires for its measurement mingle stress and strain and therefore do not allow an answer to the objectives of this review. However, assuming that all relevant aspects of stress and strain in that context are covered by the selected exposure and outcome terms, research using technostress in this differentiated manner should be found. For the complete search string see Appendix.

### Eligibility assessment

The included studies were selected according to a three-step procedure: 1) removal of duplicates and title screening, 2) abstract- and 3) full text screening. Steps 2 and 3 were carried out by two researchers independently. In case of any disagreement, the abstract or full text was reviewed by a third researcher. Screening results were discussed until agreement was reached. Eligibility criteria were established a priori. Criteria and examples of excluded texts are presented in Table 1.

**Table 1:** Study PEO inclusion and exclusion criteria

Criterion	Inclusion	Exclusion
Population	• working population	• children and youth, students, non-working population(e.g., titles like “Life satisfaction and problematic Internet use: Evidence for gender specific effects”; “Technology-based interventions for preventing and treating substance use among youth”)
Exposure	• digital technologies, work processes in a digital context • influence of technologies on employees	• use of technology for diagnostic purposes (e.g., the use of telemedicine for stroke patients like “Interactive computer-assisted program for cervical liquidbased cytology”) • use of technology for the purpose of teaching/training (e.g., introduction to new radiological technologies like “Integrating Artificial and Human Intelligence: A Partnership for Responsible Innovation in Biomedical Engineering and Medicine” or “The stress and workload of virtual reality training: the effects of presence, immersion and flow”) • focus on the use of information and communication technologies (e.g., “Impact of BYOD on organizational commitment: An empirical investigation”) • focus on the concept of technostress
Outcome	• all health/well-being outcomes in context of digital work factors	• health effects of the used technologies that do not affect the target population (e.g., improvement of schizophrenia patients through therapy applications with virtual reality like “Making monitoring ‘work’: human-machine interaction and patient safety in anesthesia” or “Optimal management of neonatal lung diseases using current technologies”)
Study	• original articles • published in peer-reviewed journals • published in English or German • no limitation of publication date	• other publication types (e.g., conference paper, editorials, project reports, non-original research such as discussion papers/reviews) • other languages

### Data extraction and evaluation

The following data were drawn from the studies: authors and year of publication, country, population (sample size and type of workplace or job task), work factors, research question, outcomes, methods, design, and results. Due to the diverse methods and concepts, no visual representation of effects or meta-analysis was possible. For this reason, we decided to use a content analysis with a subsequent categorization of the study contents as an evaluation method.

### Assessment of methodological quality

The quality of the studies was systematically checked using a tool provided by the University of Leeds, Quality Assessment Tool for Studies with Diverse Design (QATSDD; [112]). This tool is suitable when quantitative, qualitative, and/or mixed methods study designs need to be compared. All studies are evaluated against 16 criteria. The scale ranges from 0 (not at all) and 3 (complete) for each criterion. The quality assessment was done by two raters independently. In the case of disagreement, the relevant evaluation criteria were firstly discussed with a third researcher and then re-evaluated by the two raters. Diverging ratings were discussed between the two raters until consensus was reached. If no agreement could be achieved, a third rater was involved to reach the final evaluation. The interrater reliability between the two raters was assessed by the intraclass coefficient which showed a good to very good agreement (ICC = 0,871).

## Results

### Study characteristics

**Fig. 1:**
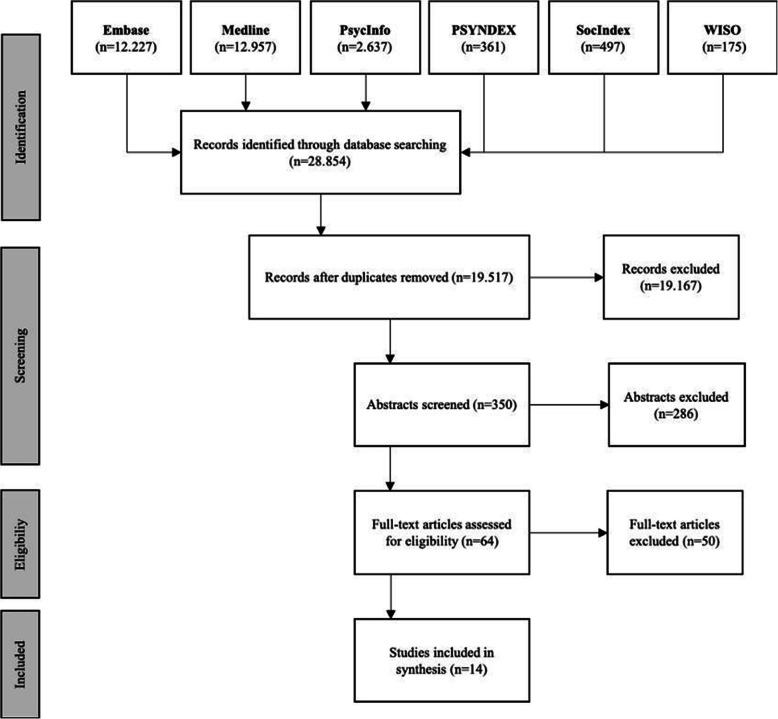
Flow diagram

In total, 28,854 studies were found. After removal of duplicates (*n* = 9337) and title screening, 350 texts remained for abstract screening. After evaluating the full texts (*n* = 64), 14 studies met the inclusion criteria and were included in this review (flow diagram in Fig. 1).

Table 2 shows the main characteristics and results of the 14 included studies. The sample size of the studies ranges from 10 to 3233 participants. All studies, to varying degrees, dealt with digital or at least technology-mediated working conditions, and investigated these in relation to health outcomes of employees. The oldest study is from 1981 and the most recent one from 2019. Eleven publications were published before the year 2000 and three between 2000 and 2019. The studied samples vary in almost equal proportions between office workers (*n* = 4), production workers (*n* = 4), and various employees (*n* = 6), for example from the banking industry or the service sector. Eleven studies were conducted in Europe, five of them from the former German Democratic Republic (GDR), two in North America and one in New Zealand. There are eight cross-sectional studies and six longitudinal studies. One study used pre-post-measurement. Five studies used a quantitative research design, one a qualitative design, and eight studies chose a mixed-methods approach. The most commonly used measurement method was questionnaires (*n* = 11), followed by work analysis (*n* = 6) and physiological measurements (*n* = 6) and interviews (*n* = 5).

**Table 2:** Characteristics of the included studies

First author, publication year, country	Sample, Size	Research Question	Design	Method	QATSDD
[4], The Netherlands	employees of automation sector, *n* = 3233	- analysis of working conditions and career prospects of 32 different occupational groups within automation personnel - identification of the risk factors for stress and strain within these occupational groups - activity group*: different occupational groups in the automation sector	- quantitative - cross-sectional	- questionnaire (adapted and extended NIPG-Questionnaire; [38])	20
[17], Sweden	“computerized” employees in administration, *n* = 42	- evaluation of work efficiency, work environment, and psychological strain before and after office automation in a participatory project - *activity group*: pre- and post-automation	- mixed - longitudinal (5 separate measures over 1,5 years)	GRID-interview; [48] physiological measurement (blood cortisol, blood pressure) questionnaire (not described; according to the authors with questions about computerization, reorganization, attitudes towards computers, current tasks and beliefs about future tasks, work content, job satisfaction, health/well-being, different symptoms of strain)	19
[19], New Zealand	employees of the service sector, *n* = 120	- analysis of how employees perceive STARA (Smart Technology, Artificial Intelligence, Robotics, and Algorithms)/job insecurity in relation to their own work and how they prepare for potential changes - analysis whether STARA-awareness/job insecurity is age dependent - analysis what possible effects STARA-awareness/job insecurity has on job and well-being outcomes (the feeling of STARA-Awareness, which “captures the extent to which employees views the likelihood of Smart Technology, Artificial Intelligence, Robotics and Algorithms impacting on their future career prospects” ([19]: p. 241))	- quantitative (plus one open-ended question) - cross-sectional	questionnaire (c*areer satisfaction*; [55]; *cynicism;* *[*77*]*; *depression;* *[*7*]*; *job insecurity;* *[*5*]*; *organizational commitment;* [82]; *STARA Awareness***;** self-developed; *turnover intentions;* [68])	21
[29], Germany (former GDR)	die-casting foundry, *n* = 25	- evaluation of flexible automation solutions compared to conventional production processes with regard to job demands, working conditions, and personality development - analysis of effects on mental well-being and job satisfaction - *activity group:* workers at conventional and flexible automated die-casting machines	- mixed - cross-sectional	interview (self-developed) questionnaire (BMS; [95], questionnaire on satisfaction with work conditions; self-developed; SAA; [1]) work analysis (TBS-K, BPA; [57])	14
[30], UK	employees of computer manufacturing company, *n* = 31	- definition and measurement of advanced manufacturing technologies (AMT) in terms of the concept of coupling; coupling is a construct that describes the degree to which two parts are connected, four variables create this construct: synchronicity, workflow rigidity, method uniformity, and slack. - identification of differences in the working conditions of different AMT-jobs - exploration of the influence of coupling on psychological well-being	- quantitative - cross-sectional	- questionnaire *(coupling:* synchronicity [31, 32]; workflow rigidity, [61]; method uniformity, [118]; Slack, Hickson, [61]; *intrinsic job satisfaction*: “Job itself intrinsic satisfaction” scale, [121]**;** *job complexity:* Perceived Intrinsic Job Charcteristics Scale, [121]**;** *mental health*: version of General Health Questionnaire, [51, 52]; *overall job satisfaction*: Job satisfaction Scale, [121]**;** *supervisory influence,* self-developed**;** *work role* breadth, self-developed)	25
[31, 32], USA	office workers, *n* = 121	- analysis of workers’ individual experiences with computers and their attitudes toward different aspects of computer work - identification of the relations of several aspects of work, in particular Video-Display-Terminal-time, and health complaints - *activity group:* employees using VDT (VDT = Video Display Terminal) to varying degrees	- mixed - longitudinal (repeated measurements on consecutive days and within one day)	- checklist (adapted and extended POMS; [80]) - physiological measurement (optometric screening procedure) - semi-structured interview following the “funnel” technique; [15]	21
[58], Germany (former GDR)	administration/Office, *n* = 240	- assessment and evaluation of VDU (visual display unit) work differing in task-content/−structure and proportion of human-machine interaction - differentiation of effects on motivation and learning opportunities - identification of task characteristics changing due to computer technology and its implementation - identification of effects of these changes on employees - *activity group:* traditional and computer-aided data entry activities with varying degrees of task completeness; activities with human-computer interaction and varying degrees of autonomy	- mixed - cross-sectional	- questionnaire/checklist (AZA; [64], BFB; [62], BMS; [95], SAA; [1]) - work analysis (TBS-GA; [57])	12
[65], Germany (former GDR)	computer screen work activities, *n* = 25	- examination of the relationship between current and long-term effects of stress caused by mental work demands - investigation whether the correlations found can be generalized and whether the consequences of stress are predictable - evaluation of influences beyond work demands (like factors outside the workplace or personal attitudes) - *activity group:* data entry via display terminal; computer-aided ticket sales; computer-aided activity for project planning of organizational processes (problem analysis)	- mixed - longitudinal (annual survey over a period of 3 years)	- interview [105], subjective job evaluation [86] - physiological measurement (e.g., heart rate and blood pressure); occupational health check-up; [122] - questionnaire/checklist (BFB; [62], BMS; [95, 96], EZ-Skala; [87]) - work analysis (occupational science checklist for computer workstations; Schönfelder and Rudolph, [108], psychological work analyses; [78], TBS-GA; [57])	18
[70], Germany	employees in areas with a high level of automation, *n* = 36	- identification of potential stressors occurring with the introduction and use of new technologies in the manufacturing industry	- qualitative - cross-sectional	- semi-structured interview (self-developed)	31
[99], Germany	operators from electric power supply system, *n* = 50	- evaluation of reliability of human operators in highly automated systems using intra- and interindividual differences in physiological and psychological data for the identification of unreliability and action failures - *activity group:* operators in the electroenergy network with the different sub-activities “planned intervention”, “monitoring”, “fault processing”.	- mixed - longitudinal (repeated measurements within one day of examination)	- physiological measures (heart rate, blood pressure) - questionnaire ([98], EZ-Skala; [87]) - work analysis (TBS-GA; [108])	23
[109], Germany (former GDR)	plant operators, *n* = 119	- evaluation and comparison of physical and mental strain during activities in the automotive industry with different levels of automation - *activity group* plant operators in vehicle body construction; plant operators in automated final assembly; plant operators in driverless transport systems; assembly workers in body and vehicle final assembly	- mixed - cross-sectional (physiological measurements repeated in the course of a shift)	- physiological measurements (cardiopulmonary capacity, physical activity, oxygen expenditure, biochemical parameters (e.g., adrenalin), heart rate) - work analysis (occupational science survey procedure for activity analysis; [107])	18
[111], Germany (former GDR)	plant operators at a metal factory, *n* = 10	- evaluation of psychophysical stress and resulting health risks through changes in work content and extended work shifts in automated production processes - *activity group:* early shift 8 h; early shift 12 h; late shift 8 h; late shift 12 h	- mixed - longitudinal (repeated in the course of a shift)	physiological measurements (heart rate) questionnaire/checklist (rating scale), EZ-Skala; [87]) work analysis (workday recording, occupational science survey procedure for activity analysis; [107], objective/subjective stress screening, TBS; Hacker et al., [57])	12
[114], USA/Canada	female clerical workers, *n* = 1032	- examination of the relationship between extent of video display terminal (VDT) use and employees’ perceptions of physical work environment, job characteristics and health/well-being - analysis of differences between health symptoms and job characteristics of supervisors and non-supervisors - *activity group*: part-day typist; all-day typist; clerical worker; part-day VDT user; all-day VDT user	- quantitative - cross-sectional	questionnaire (self-developed and according to the author with questions about physical environment, job characteristics, psychological/physical health, and job satisfaction)	20
[117], Sweden	bank employees, *n* = 151	- analysis of bank employees’ evaluation of the role of digitization in their daily work - analysis of bank employees’ evaluation of the role of digitization and its effects on well-being - exploration of the interaction between digitization and organizational culture (either individualistic or collectivistic) and its effects on well-being - examination of the influence of age, organizational tenure, and position	- quantitative - cross-sectional	- questionnaire *(job satisfaction*: Job Satisfaction Scale; [3]; *life balance*: Affect Balance Scale; [16]; *life satisfaction*: Satisfaction with Life Scale; [37]; *use of digital tools;* self-developed; *organizational culture*: seven-adjectives-Scale; [26]))	22

*activity group – classification within the study according to different activities; further explanation in section *clustering of work factors, health effects and activity groups*; NIPG – Questionnaire on Work and Health (NIPG-TNO; [38]); GRID-Interviews [48]; self-developed – according to the authors; BMS – BeanspruchungsMessSkalen [95, 96], SAA – Fragebogen zur subjektiven Arbeitsanalyse [1], TBS-GA – Tätigkeitsbewertungssystem für geistige Arbeiten [57], BPA – Analyse und Bewertung der persönlichkeitsfördernden Wirkung von Arbeitsaufgaben, POMS – Profile of Mood States [80], AZA – Zufriedenheit mit der Arbeit [64], BFB Beschwerdefragebogen [62], EZ-Skala [87], TBS-GA – Tätigkeitsbewertungssystem für geistige Arbeiten [108]

### Quality assessment

All studies were evaluated with the QATSDD tool. The reviewed studies scored between 12 and 31 (M = 19.71, SD = 4.87), which represents 27–69% of the achievable maximum value. The ratings of the individual studies are shown in Table 2. On a scale from 0 (“not at all”) to 3 (“complete”), most of the studies gave an almost complete description of the research setting (M = 2.71; SD = 0.45). The objective of the study was also moderately explicitly stated (M = 2.0; SD = 0.96). Both the fit between the stated research question and the method of data collection of the quantitative studies (M = 2.0; SD = 0.75) and the fit between research question and method of analysis (M = 2.0; SD = 0.53) moderately met the criteria. In the qualitative methods, the fit between the research question and the format and content of data collection was rather low (M = 1.21; SD = 1.01). A similar assessment was made for the description of an explicit theoretical framework (M = 1.21; SD = 1.25). Criteria of the data collection process (M = 1.92; SD = 0.82) and the rationale for choice of data collection tools (M = 1.5; SD = 0.90) were only very slightly met in most studies. The justification for the selected analytical method was mostly rated between “not at all” and “very slightly” (M = 0.78; SD = 0.67). Criteria for detailed recruitment data were met only slightly (M = 1.07; SD = 0.70). Evidence of sample size considered (M = 0.5; SD = 0.90) and the representativeness of the target group (M = 0.85; SD = 0.86) did not meet the criteria at all. The same applies to the reliability and validity of measurement tools in quantitative studies (M = 0.78; SD = 1.14) as well as to the reliability of the analytical process of qualitative studies (M = 0.35; SD = 0.89). The discussion of strengths and limitations was mostly not mentioned at all (M = 0.71; SD = 0.79). In none of the studies included in the review, information on user involvement in the study design was given.

### Clustering of work factors, health effects and activity groups

To summarize the studies, we first clustered all identified working conditions as work factors (independent variables) and outcomes as health factors (dependent variables).

As a basic framework for the clusters of *work factors,* we used the “Recommendations for the implementation of risk assessment of mental stress” (European Commission: Guidance on risk assessment at work, [42]):

- *Cluster: cognitive demands/work content (task).* This includes demands and working conditions like decision latitude, task variability, or qualification.
- *Cluster: social factors.* This refers to the social relationships among colleagues and/or superiors, which include, for example, conflicts and support situations, number of contacts, lack of feedback, and leadership.
- *Cluster: organizational factors*. Organizational factors include mainly the topics working time and workflow (e.g., time pressure/high workload) but also communication and cooperation possibilities of employees (e.g., isolated workstations) as well as information on salary and career opportunities.
- *Cluster: environmental factors/working tools*. Environmental factors include workplace and work equipment design as well as ergonomic and physical/chemical factors such as light or noise. Working tools incorporate all technologies used. The type of application like the reason for use, for example for information retrieval or for work optimization, are also included in this cluster. In addition, this term covers not only the devices, applications, and tools themselves, but also effects on the work system as technically caused interruptions and disturbances or, for example, Corbett’s coupling term [30].

The majority of studies (*n* = 10) do not use the examination of single work factors as a starting point but constellations of work factors in the context of specific work activities. Thus, it was necessary to set up so-called *activity groups* as a further category of clusters. Three activity groups were aggregated:

- *activity group 1 (ag1):* Extent of technology use, for example display screen activities differentiated by duration of display screen use (*n* = 5) [4, 29, 32, 109, 114]
- *activity group 2 (ag2):* Working conditions before and after automation/computerization (*n* = 1) [17]
- *activity group 3 (ag3):* Level and extent of mental tasks, e.g., “monitoring” vs. “control” activities (*n* = 4) [58, 65, 99, 111].

In the category *health factors,* the following clusters were formed:

i) *Motivation and satisfaction:* Concepts like motivation, organizational commitment, or satisfaction in all their facets as well as turnover intention.
ii) *Reduced well-being/affective symptoms:* Psychological symptoms like irritation and feelings of stress as well as depression and anxiety; fatigue, monotony, and saturation are included.
iii) *Physiological parameters and somatic complaints:* All outcomes of physical examinations such as blood pressure, heart rate or somatic complaints like musculoskeletal or eye symptoms.

Table 3 provides an overview of all work factors and health factors found in the studies, grouped according to these clusters.

**Table 3:** Focus and key work and health factors

*First author, publication year, country*	*Main focus of the study*	
	***work factors***	***health factors***
Andries*, 1991 [4], The Netherlands	Focus: groups of different occupations in the automation sector - differentiation of risk factors for health	
	*(a) cognitive demands/work content:* challenge of the job (e.g., engaging, offering pleasure), qualification (e.g., education, experience, training), autonomy *(b) social factors:* quality of leadership, contacts with colleagues *(c) organizational factors:* workload (e.g., working hours), hectic working conditions (time pressure, unexpected events), salary and prospects	*(ii) reduced well-being/affective symptoms:* mental strain (e.g., feeling tense, nervous or agitated) *(iii) physiological parameters/somatic complaints:* health complaints, headaches, sleep
Brenner*, 1995 [17], Sweden	Focus: participatory introduction of computerization - changes in working conditions and health effects	
	*(a) cognitive demands/work content:* qualification, responsibility, task variety, reorganization *(b) social factors:* contacts with fellow-workers and supervisors *(c) organizational factors:* computerization, beliefs about how future tasks would appear, workload *(d) environmental factors/working tools:* computer disturbances	*(ii) reduced well-being/affective symptoms:* mental strain, experience with and attitudes toward computers, nervousness *(iii) physiological parameters/somatic complaints:* somatic symptoms (sleep, heart, fatigue, stomach, musculoskeletal), physiological measures (e.g., cortisol, blood pressure)
[19], New Zealand	Focus: STARA (Smart Technology, Artificial Intelligence, Robotics, and Algorithms)-Awareness - impact on job and well-being outcomes	
	*(c) organizational factors:* job insecurity/STARA-Awareness	*(i) motivation and satisfaction:* career satisfaction, organizational commitment, turnover intention *(ii) reduced well-being/affective symptoms:* depression, cynicism
Claussner*, 1989 [29], Germany (former GDR)	Focus: different degrees of automation - consequences for health-promoting work design	
	*(a) cognitive demands/work content:* decision latitude, task variability, transparency, responsibility, cognitive demands *(b) social factors:* social structure (social support, feedback) *(c) organizational factors:* workload (quantitative and qualitative overload), human-machine-division of labor, workflow *(d) environmental factors/working tools:* environmental conditions, usability of technologies	*(i) motivation and satisfaction:* satisfaction with different working conditions (e.g., work design, technical equipment, skill use, division of labor) *(ii) reduced well-being/affective symptoms:* strain, monotony, saturation
[30], UK	Focus: coupling in the context of advanced manufacturing technologies (AMT) - effects on well-being and work demands	
	*(a) cognitive demands/work content:* job complexity, work role breadth *(b) social factors:* supervisor influence *(d) environmental factors/working tools:* technological coupling (synchronicity, workflow rigidity, method uniformity, slack)	*(i) motivation and satisfaction:* intrinsic job satisfaction, overall job satisfaction *(ii) reduced well-being/affective symptoms:* mental health (e.g., anxiety, depression, low self-esteem)
Dainoff*, 1981 [32], USA	Focus: physical/mental stress and other effects of computer work (e.g., job pressure) as a function of VDT time	
	*(a) cognitive demands/work content:* task variability *(b) social factors:* quality of leadership, atmosphere with coworkers, customers and supervisors *(c) organizational factors:* pressure, pay, benefits, job insecurity *(d) environmental factors/working tools:* ergonomic comments (e.g., light, noise, temperature, workplace arrangement), interruptions, problems with computer system (e.g., slow response time)	*(ii) reduced well-being/affective symptoms:* mental stress (tension, mental strain), general fatigue (very tired, exhausted, drained after work) *(iii) physiological parameters/somatic complaints:* visual performance (measures of acuity, lateral phoria, and vertical phoria, visual strain (e.g. blurred vision)), physical stress (headaches)
Hacker*, 1985 [58], Germany (former GDR)	Focus: different task-content/−structure and proportion of human-machine interaction in different VDU work associations with task characteristics and strain	
	*(a) cognitive demands/work content:* autonomy, task variability, transparency, qualification, excessive demands, learning requirements *(c) organizational factors:* cooperation requirements and opportunities, information on hardware/software	*(i) motivation and satisfaction:* job satisfaction, motivation *(ii) reduced well-being/affective symptoms:* psychological complaints, experienced monotony, saturation, stress *(iii) physiological parameters/somatic complaints:* physical complaints
Jackisch*, 1989 [65], Germany (former GDR)	Focus: mental demands during VDU-work - predictability of long-term health effects	
	*a) cognitive demands/work content:* cognitive demands	*(i) motivation and satisfaction:* job satisfaction, behavioral parameter (e.g., performance) *(ii) reduced well-being/affective symptoms:* current well-being, experienced monotony, saturation, stress *(iii) physiological parameters/somatic complaints:* physiological parameters (heart rate and blood pressure), complaints, sick leave
[70], Germany	Focus: human-machine-interaction - stress	
	*(a) cognitive demands/work content:* deskilling, qualification requirements, situation awareness *(c) organizational factors:* general evaluation of human-machine interaction *(d) environmental factors/working tools:* technical problems (e.g., software/hardware problems) usability (e.g., self-descriptiveness)	*(ii) reduced well-being/affective symptoms:* stress
Rau* (1996) [99], Germany	Focus: human reliability in complex automated systems and associated health effects	
	*(a) cognitive demands/work content:* responsibility, cognitive demands	*(i) motivation and satisfaction:* motivation *(ii) reduced well-being/affective symptoms:* mental tension, emotional state, locus of control, current intrinsic states (e.g., ready to exert, tensioned, self-assured) *(iii) physiological parameters/somatic complaints:* heart rate, blood pressure
Rutenfranz*, 1989 [109], Germany (former GDR)	Focus: changes in physical, mental, and emotional strain through automation	
	*(a) cognitive demands/work content:* complexity, responsibility, variability, cognitive demands *(c) organizational factors:* breaks *(d) environmental factors/working tools:* disruptions	*(iii) physiological parameters/somatic complaints:* biochemical parameters (adrenalin/noradrenalin), heart rate, energy expenditure, cardiopulmonary performance (physical examination, bicycle ergometer)
Seibt*, 1988 [111], Germany (former GDR)	Focus: shift work - health effects	
	*(a) cognitive demands/work content:* task content, action control *(b) social factors:* social integration *(d) environmental factors/working tools:* aggravating conditions (e.g., noise)	*(i) motivation and satisfaction:* readiness to make an effort *(ii) reduced well-being/affective symptoms:* experienced strain (initiative, self-confidence, emotional tension, fatigue) *(iii) physiological parameters/somatic complaints:* heart rate
Stellman*, 1987 [114], USA/Canada	Focus: extent of video display terminal usage - description of job characteristics with analyses of health effects	
	*(a) cognitive demands/work content:* task variability, decision latitude, repetitious work, understanding of work process, learning new things, work “makes sense”, cognitive demands *(c) organizational factors:* workload *(d) environmental factors/working tools:* physical characteristics of the office (e.g., ergonomic stressors, air quality stressors, privacy)	*(i) motivation and satisfaction:* job satisfaction, office satisfaction *(ii) reduced well-being/affective symptoms:* psychological symptoms (irritation, anxiety, depression, hopelessness) *(iii) physiological parameters/somatic complaints:* health symptoms (eye-, musculo-skeletal-, gastrointestinal-, respiratory- symptoms)
[117], Sweden	Focus: perception of digitalization - the effect on subjective well-being of bank employees	
	*(b) social factors:* organizational culture *(d) environmental factors/working tools:* digitalization (degree of use of digital tools, subjective experiences associated with the use of digital tools)	*(i) motivation and satisfaction:* job satisfaction, life balance, life satisfaction

(*studies categorized by activity group)

### Study findings

The main focus in reviewing the included studies was to assess work factors associated with digitally connected work, interrelations of work factors and relationships between work factors and health factors. As not all studies provide statistical evidence or report detailed data, we had to restrict the analysis to frequency counts. In a first step we summarize the total counts for every cluster irrespective of the additional activity groups categorization. Since all work factors or health outcomes associated with digitally connected work are relevant to the research question, all mentions were counted (*cluster (a) cognitive demands / work content (−task)* (*n* = 102), *cluster (d) environmental factors/working tools* (*n* = 73)*, cluster (c) organizational factors* (*n* = 46), *cluster (b) social factors* (*n* = 17); *cluster (iii) physiological parameters and somatic complaints* (*n* = 72), *cluster (ii) reduced well-being and affective symptoms* (*n* = 30), *cluster (i) motivation and satisfaction* (*n* = 29)).

After a paragraph on the theoretical framework of the identified studies, we present the number of reported associations between the work factor/activity group clusters and the health clusters aggregated over all studies. Associations that were actually only reported but not described in detail were also included here, as the very combination of two characteristics is interesting in this context. Additionally, tabulated summaries of statistically tested associations in aggregated verbal form are provided. Remarkable single findings are reported following each descriptive section. Results are always reported with their publication date as there is a large time gap between the oldest and most recent publication. The results are described in the corresponding text in descending order according to the counts.

### Theoretical framework of identified studies

As the quality assessment shows, the theoretical background is rarely found in the texts and, if so, only briefly addressed. Due to the scope of the review, most studies are rooted in work and organizational psychology. All GDR studies follow the theoretical principles of action regulation theory and pursue the goal of a work design that promotes personality [29, 58, 65, 99, 109]. Hacker and Schönfelder [58], Körner et al. [70] and Stellman et al. [114] substantiate the results with reference to the Job Demand Control Model by Karasek [66]. Brenner et al. [17] based their study on the theory of stress of Lazarus [72] and interpret differences in the expression of stress with different attitudes and other individual factors [73]. They cite the Vitamin model [120] to explain environmental (working) conditions that affect mental health differentially and to varying degrees. To explain the relationship between increased demands and effects on both psychological and physiological stress, they refer to the theoretical principles of Frankenhaeuser [46, 47]. Corbett [30] refers in the interpretation of his results to the theory of Hackman and Oldham [59].

### Associations between work factors and health factors

Table 4 shows the associations between work factors and health factors regarding the number of associations with statistical tests and verbally aggregated direction of effects.

**Table 4:** Summary of the associations between work factors and health factors

***First Author***	***Work factor clusters (iv)***	***Health outcome cluster (dv)*** Number of reported associations in total*	***Number of reported direct associations with statistical analysis***	***Number of reported associations without statistical analysis***	Direction of effects**	***Number of reported effects with work/health variables as moderator /mediator/ control***
[30];	Cognitive demands / work content	Motivation and satisfaction (4)	4	0	inconclusive	
[17];		Reduced well-being / affective symptoms (5)	3	2	not significant	
[70]		Physiological parameters and somatic complaints (4)	4	0	significant positive	
[30];	Social factors	Motivation and satisfaction (4)	2	0	not significant	2
[117]		Reduced well-being / affective symptoms (1)	1	0	not significant	
		Physiological parameters and somatic complaints (0)	0	0		
[19],	Organizational factors	Motivation and satisfaction (3)	0	0		3
[32],		Reduced well-being / affective symptoms (4)	2	0	significant positive	2
[17]		Physiological parameters and somatic complaints (3)	3	0	significant positive	
[30];	Environmental factors / working tools	Motivation and satisfaction (13)	13	0	inconclusive	
[117];		Reduced well-being / affective symptoms (11)	3	8	significant positive	
[32], [70]		Physiological parameters and somatic complaints (0)	0	0		

**Fig. 2:**
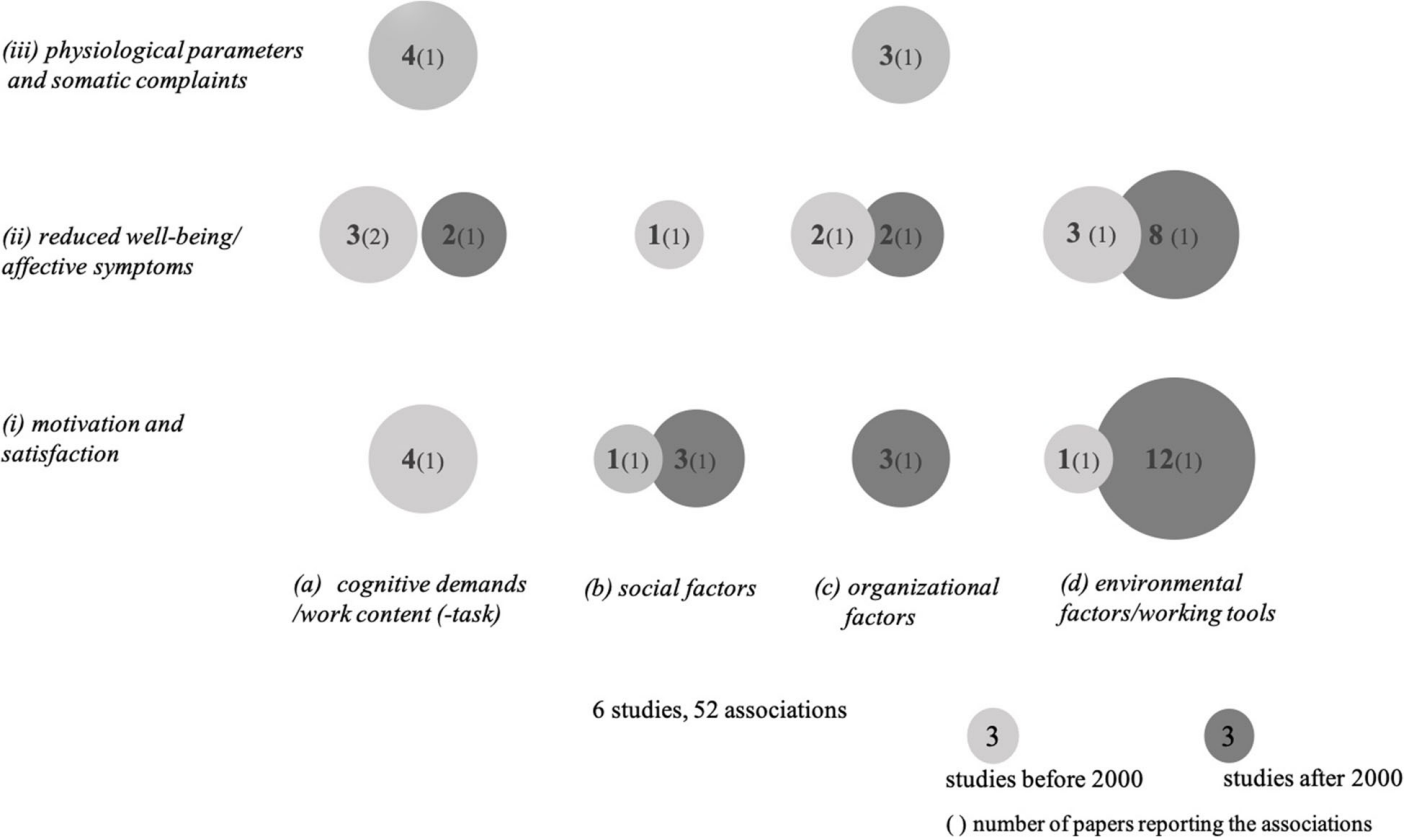
Frequency of associations between work factors and health factors

* number of papers represented by the counts: see Fig. 2

** summary assessment independent of underlying construct; significant negative/significant positive = if 75% of reported associations are significant negative/significant positive and the rest is not significant; all other combinations: inconclusive; not significant = no significant association at all

Figure 2 presents all associations between work factor clusters and health clusters investigated in the studies irrespective of which part of the publication (results or discussion) they are reported in. The associations also include results from activity group studies if they investigated distinct work and health factors.

#### Cluster (d) environmental factors/working tools

This cluster is most frequently associated with *cluster (i) motivation and satisfaction* with 13 counts. The main focus is on the outcome job satisfaction. All 12 results after 2000 are exclusively found in Umans et al. [117]. They examined each of three outcome variables (job satisfaction, life satisfaction, life balance) with four work factors, which are differentiated by the intended use of digital tools. They found overall significant positive associations; especially the use of digital tools for the purpose of work optimization has a positive relation to all three satisfaction facets. Furthermore, life satisfaction has a significant positive correlation with the use of digital tools for information management. The older study in this context, Corbett [30], described a significant negative influence of coupling on intrinsic job satisfaction.

The second often investigated relationship is this cluster in conjunction with *cluster (ii) reduced well-being/affective symptoms* [30, 32, 70]. Corbett [30] showed a significant positive association between coupling and mental health. In their qualitative interview study, Körner et al. [70] identified a connection between technical interruptions and software, hardware, or usability problems and perceived stress. Dainoff et al. [32] showed that there is a significant positive correlation between VDT-related lighting problems and stress.

In sum, negative (e.g., stress) as well as positive health effects (e.g., job satisfaction) are related with environmental factors and the application of technological tools.

#### Cluster (a) cognitive demands/work content

Relations with *cluster (ii) reduced well-being/affective symptoms* were investigated by three studies [17, 30, 70]. In the Corbett study [30], multiple regression analysis reveals no significant association of job complexity or role breadth with mental health. Brenner et al. [17] show that after computerization, a higher experienced level of job qualification is significantly positively correlated to a high frequency of several psychosomatic symptoms like nervous symptoms. Körner et al. [70] point out that insufficient qualification in handling digitalized hard- and software seem to be related to stress.

Relations with *(i) motivation and satisfaction* are only considered by Corbett [30] who shows that job complexity is positively related to overall job satisfaction.

The only study that examined variables in the context of *cluster (iii) physiological parameters and somatic complaints* is Brenner et al. [17]. They report positive associations between qualification, in the sense of more qualified work after automatization, and a high frequency of sleep-, heart-, and fatigue- symptoms.

In sum, *cluster (a) cognitive demands/work content* is dominated by different aspects of qualification. Two studies [17, 70] assume that there is an increased need for qualification when using digital technologies. Insufficient qualification and skills can lead to psychological and physiological stress. The job complexity factor is generally rated positively.

#### Cluster (c) organizational factors

This cluster has most associations with health effects of *cluster (ii) reduced well-being/affective symptoms.* Dainoff et al. [32] report a weak but significant positive correlation between job pressure and mental stress, and also between job pressure and fatigue in the sense of tiredness and exhaustion. Brougham et al. [19] show that when job insecurity is prevalent among employees, there is a positive significant association with depression and cynicism.

Second most frequently reported, in one study, are associations between *cluster (c) organizational factors* and *(iii) physiological parameters and somatic complaints.* Brenner et al. [17] found positive significant correlations between workload and sleep-, heart-, and musculoskeletal symptoms.

Only Brougham et al. [19] investigate outcome variables which are related to *cluster* (*i) motivation and satisfaction*. They report that the overall perception of job insecurity, in the sense that technology replaces jobs, is low. This is reflected in a significant negative correlation between job insecurity and both organizational commitment and career satisfaction. Consequently, they find a positive significant correlation between job insecurity and turnover intention.

#### Cluster (b) social factors

Variables from *cluster (b) social factors* were the most scarcely considered work factors in the context of health effects [30, 117]. Umans et al. [117] examined the moderating effect of organizational culture on job and life satisfaction and on life balance, all assigned to *cluster* (*i) motivation and satisfaction*. The only significant effect was found with life balance. A collectivist organizational culture was found to moderate the effect of the use of digital tools for work optimization on life balance. Furthermore, a higher collectivist organizational culture was found to result in a better life balance. In the study by Corbett [30], no significant relationship was found between supervisor influence and different satisfaction variables.

There was also no significant correlation between supervisor influence and mental health in the Corbett study [30], which was assigned to *cluster (ii) reduced well-being/affective symptoms*.

### Associations between activity groups and health factors

Table 5 shows the associations between activity groups and health factors regarding the number of associations with statistical tests and verbally aggregated direction of effects.

**Table 5:** Summary of the associations between activity groups* and health factors

***First author, publication year***	***Activity group cluster (iv)***	***Health outcome cluster (dv)*** Number of reported associations in total**	***Number of reported direct associations with statistical analysis***	***Number of reported associations without statistical analysis***	Direction of effects ***	***Number of reported effects with work/health variables as moderator /mediator/ control***
[4, 29]; [109]; [114]	activity group 1 (extent of technology use)	Motivation and satisfaction (12)	11	1	inconclusive	0
		Reduced well-being / affective symptoms (9)	2	7	not significant	0
		Physiological parameters and somatic complaints (43)	34	9	significant positive	0
[17]	activity group 2 (before and after automation/computerization)	Motivation and satisfaction (0)	0	0		0
		Reduced well-being / affective symptoms (1)	1	0	not significant	0
		Physiological parameters and somatic complaints (6)	6	0	significant positive	0
[58, 65, 99 111]	activity group 3 (level and extent of mental tasks)	Motivation and satisfaction (7)	3	4	inconclusive	0
		Reduced well-being / affective symptoms (11)	8	3	significant positive	0
		Physiological parameters and somatic complaints (8)	4	4	significant positive	0

* this table refers to group comparisons and the association was reported with the group with the highest extent of technology use, the group after automation, or the group with the highest level of mental tasks

**Fig. 3:**
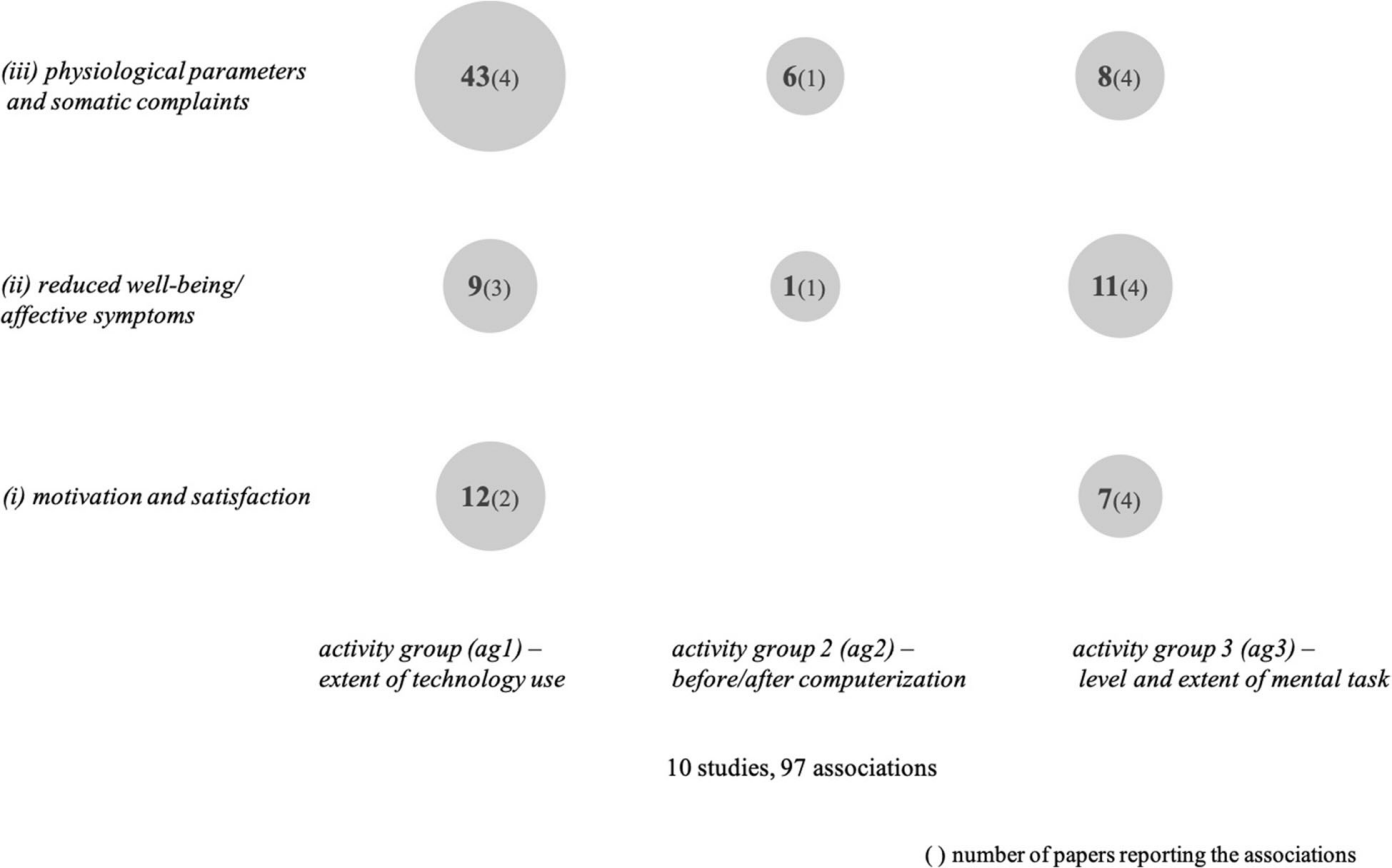
Frequency of associations between activity groups and health factors

** number of papers represented by the counts: see Fig. 3

*** summary assessment independent of underlying construct; significant negative/significant positive = if 75% of reported associations are significant negative/significant positive and the rest is not significant; all other combinations: inconclusive; not significant = no significant association at all

Figure 3 summarizes all mentioned associations between activity group clusters and health factors irrespective of which part of the publication (results or discussion) they are reported in.

All ten studies with activity groups report associations with health factors (Table 3).

#### Activity group 1 (ag1) - extent of technology use

*(Ag 1)* shows most (43) associations with health outcomes from *cluster (iii) physiological parameters and somatic complaints.* With 25 associations, the study by Dainoff et al. [32] contributes most to this category with the results of a large number of differentiated eye examinations by VDT users. However, they could not find any significant effects depending on time of day. Visual symptoms were also examined in the study by Stellman et al. [114]. They show that those who work full-time on a PC have increased eye complaints. Considering other somatic symptoms such as gastrointestinal or respiratory symptoms, no differences could be observed with respect to varying degrees of technology use. Rutenfranz et al. [109] show that those who use automated tools the most have both a higher heart rate and higher blood pressure.

With 5 out of 9 associations, *cluster (ii) reduced well-being/affective symptoms* is characterized by the study of Andries et al. [4] who found that workers in middle management occupations in the automation sector often feel tense and that occupations with a demanding and responsible character often feel agitated. Dainoff et al. [32] show that the correlation between VDT-time and extent of stress is low and not statistically significant.

*Cluster (i) motivation and satisfaction* is dominated by the outcome job satisfaction. Claussner and Müller [29] report that work at automated machines is associated with higher satisfaction with mental and physical demands. Stellman et al. [114] found that job satisfaction among all-day VDT-users was lower compared to part-day VDT-users. Satisfaction with the office and the environment was very high among all-day VDT-users and lower among part-day VDT-users and part-day typists.

In sum, these associations highlight the fact that the extent of technology has a significant impact on the mental and physical health of employees.

#### Activity group 2 (ag2) - before/after computerization

The measurements for *(ag2)* [17] after computerization further show a positive association between workload and the frequency of sleep-, heart-, and fatigue-symptoms as well as with nervous symptoms, that is, after computerization nervous and fatigue symptoms were higher than before. Additionally, they found increased cortisol levels during the introduction of computerization but conclude that this was rather an (early) effect of activation due to job enrichment than of negative stress.

#### Activity group 3 (ag3) - level and extent of mental task

In *(ag3)*, all three health clusters are researched almost to an equal extent by all four papers [58, 65, 99, 111]. The focus of *cluster (ii) reduced well-being/affective symptoms* is on fatigue, monotony, and saturation. Hacker and Schönfelder [58] show that in activity groups with less challenging work contents, the values of fatigue and saturation range in scores that indicate persistently reduced work efficiency. In a longitudinal assessment, Jackisch et al. [65] found that differences in (health-)effects of strain (including fatigue, monotony, saturation) are activity-specific. Nevertheless, the longitudinal analysis also showed that not one single factor but a multifactorial set of conditions is causative for long-term effects of strain. Rau [99] shows that the level of demand of the activity is essential for the judgement of the emotional condition. This is more negative if the highest demand level is present. This group also reports lower feelings of control.

In *cluster (i) motivation and satisfaction,* Hacker and Schönfelder [58] find that satisfaction with the task and qualification is lower in activity groups with less challenging work content. Nevertheless, the activity group with the most challenging work content (managers) reports very low satisfaction with both the tasks and the demands. Hacker and Schönfelder [58] attribute this to a very high level of responsibility and time pressure associated with this activity group. Seibt et al. [111] show a decrease in work engagement only for the activity group with an extended morning work-shift.

Variables of *cluster (iii) physiological parameters and somatic complaints* were mainly heart rate and blood pressure measurements and physical complaints. Hacker and Schönfelder [58] report psychophysical results only for one activity group. They observed statistically significant fewer complaints in the activity group with a more challenging work content. Rau [99] proved that differences in heart rate and blood pressure were associated with the requirement level of the individual activity. Activities that dealt with malfunctions or performed interventions were associated with significantly higher blood pressure and heart rates than monitoring activities. Seibt et al. [111] found no statistically significant correlation between activity groups and heart rate concerning duration of the activity and time of the day. Jackisch et al. [65] report aggregated data; blood pressure and somatic complaints are parameters from the set they used to differentiate the effects of strain (see associations with *cluster ii*).

In sum, results suggest that the most complex activities are reflected in the highest values of psychophysiological values such as heart rate, blood pressure and lesser emotional well-being.

### Interrelation of work factors and associations with activity groups

**Fig. 4:**
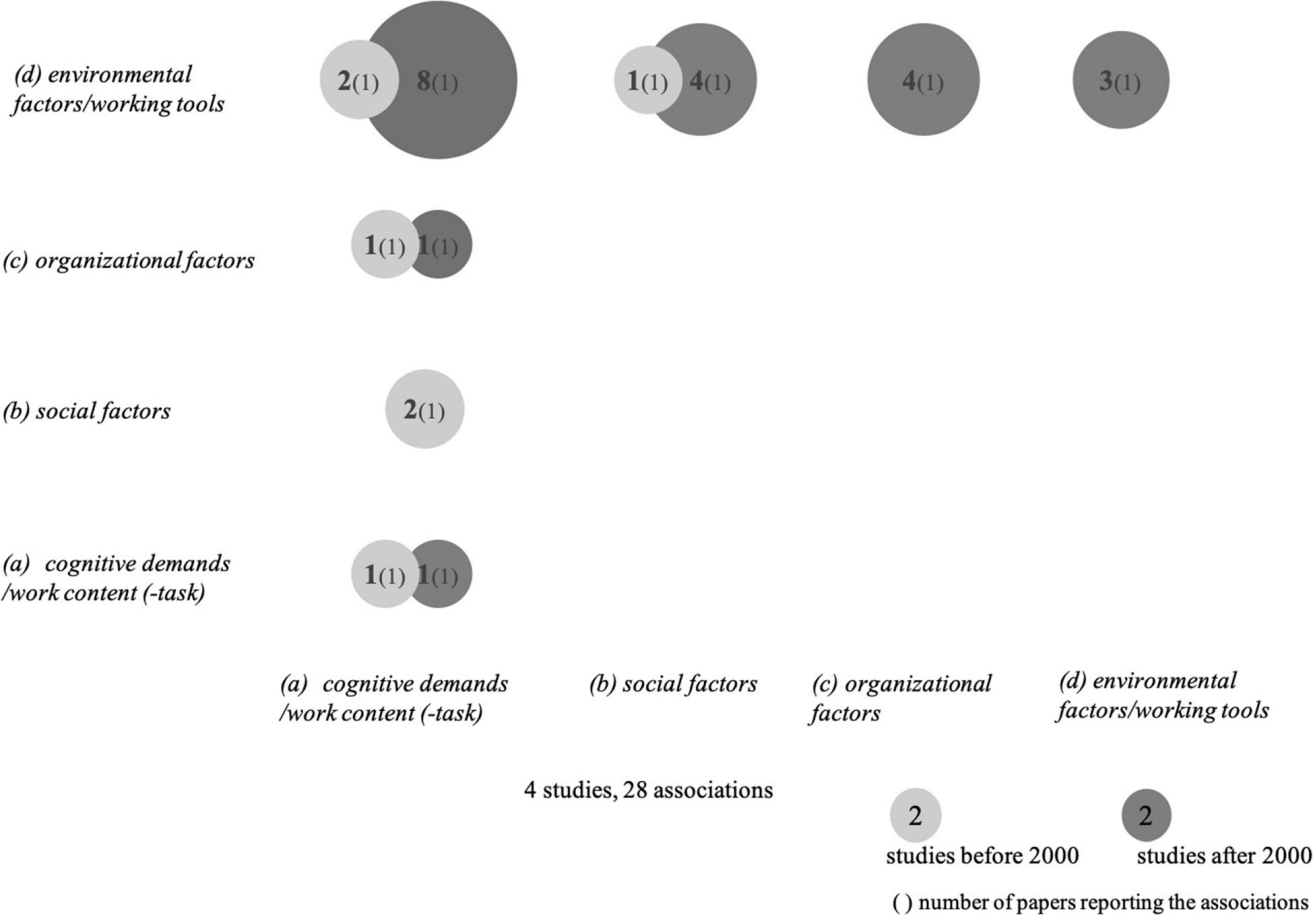
Frequency of interrelations between work factors

Figure 4 summarizes the interrelations between the work factor clusters including results from activity group studies if they investigated interrelations between distinct work factors.

#### Cluster (d) environmental factors/working tools

Associations with *cluster (a) cognitive demands/work content (−task)* are considered most often. Körner et al. [70] conclude from the analysis of their interviews that human-machine interaction can lead to many positive effects resulting from the acceleration of work processes, more precision in the manufacturing process and the reduction of undetected errors. They also point out that frequently changing and more and more complex systems demand intensified qualification. Corbett [30] examined the work factors job complexity and role breadth, both assigned to *cluster (a) cognitive demands/work content (−task)*, in connection with coupling and did not find significant associations. For associations with *cluster (b) social factors*, Corbett [30] shows a positive significant correlation between supervisor influence and coupling. Umans et al. [117], who used organizational culture as a moderation variable, demonstrate that a higher degree of a collectivist organizational culture improves the relationship between the use of digital tools for work optimization and life balance.

Associations between *clusters (d) environmental factors/working tools* and *(c) organizational factors* and within *(d) environmental factors/working tools* were investigated only by Körner et al. [70]. They conclude that time pressure is often related to technical problems and hard- and software problems and that interdependencies can aggravate this pressure. They also report a connection between low transparency of automated systems and resulting time pressure. Within the *cluster (d) environmental factors/working tools*, Körner et al. [70] show a relation between technical errors and subsequent interruptions.

In sum, results of this analysis of interrelations of work factors show how strongly working conditions affect each other. If the processes within the *environmental tools/working tools* do not work, they can block each other and thus delay the entire work process. In order to be able to handle this, certain qualifications are required.

#### Cluster (a) cognitive demands / work content (−task) with further work factors

Two studies have investigated relations within *cluster (a)* [30, 70]. Corbett [30] shows a positive significant correlation between job complexity and role breadth. Körner et al. [70] report that situation awareness and qualification were rarely linked by employees.

Corbett [30] was the only study to investigate the relationship between *cluster (a)* and *(b) social factors*. He found no significant associations between supervisor influence and role breadth, or between supervisor influence and job complexity.

For the association between *cluster (a)* and *cluster (c) organizational factors*, Brenner et al. [17] show a significant positive correlation between workload and qualification. Körner et al. [70] report positive effects of human-machine interaction on flexibility in the work process*.*

In sum, the studies of this cluster combination show that the complexity to be managed as well as the qualification of employees are essentially related to the successful management of workload.

**Fig. 5:**
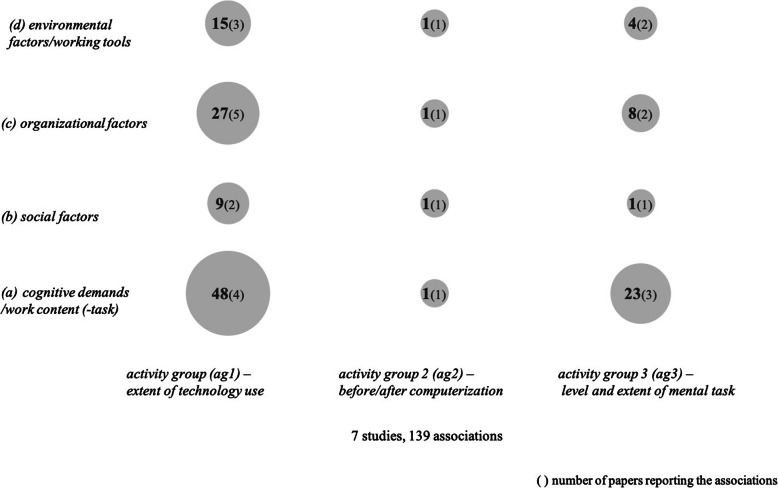
Frequency of associations between activity groups and work factors

Figures 5 summarizes the associations between the work factor clusters and activity groups.

#### Activity group 1 (ag1) - extent of technology use

Four studies from *(ag1)* examined work factors from *cluster (a) cognitive demands/work content (−task)*. Andries et al. [4] showed variations in task variability, autonomy, and qualification for different activity groups in the automation sector. Claussner and Müller [29] considered task variability, degrees of freedom, responsibility, cognitive and physical demands of workers at conventional and flexible automated die-casting machines. Overall, he found that work at the automated machine is more beneficial in terms of personality development as it offers for example a greater task variability and more latitudes, more responsibility, and fewer physical demands. Rutenfranz et al. [109] show different degrees of complexity depending on the automated task area. The highest degree of complexity was found for those with the highest requirements in handling automated work orders. In Stellman et al. [114], the group which used digital tools to the greatest extent (all-day VDT user) had the lowest decision latitude, transparency, variability, and learning requirements.

Summarizing the results, it seems as if the *extent of technology use* is unanimously seen as a factor changing the cognitive demands of work – sometimes resulting in higher cognitive demands with higher qualification demands but sometimes also in lower cognitive demands, which means a dequalification of employees.

Associations of *(ag1)* with *cluster (c) organizational factors* predominantly contained time pressure and workload. Dainoff et al. [32] report that the VDT-time (as group defining feature) had a significant negative correlation with job pressure. Employees who spent the least time working before the screen reported the highest job pressure. In contrast, Stellman et al. [114] report the highest workload for all-day VDT users. Rutenfranz et al. [109] show that the different degrees of activity can be seen above all in the demands of experiencing time pressure. This results from a responsibility for errors and the resulting possible delays in the process.

The combinations of *(ag1) - extent of technology use* and *cluster (d) environmental factors/working tools* are limited to ergonomic aspects. For example, VDT-time and light have a highly positive significant correlation [32]. Stellman et al. [114] report the highest levels of ergonomic stressors for the group with the highest level of technology use.

Two studies investigated associations with *cluster (b) social factors*. The focus is on feedback [29] and on leadership and corporate culture [4], but both do not report results.

#### Activity group 2 (ag2) - before/after computerization

Brenner et al. [17] is the only study covering *(ag2) - before/after computerization*. They considered one connection to every cluster of work factors. Related to *cluster (a) cognitive demands/work content (−task),* work after computerization is perceived as more qualified than before automation. Concerning *cluster (c) organizational factors,* technology-induced interruptions result in higher workload. Handling these interruptions requires more attention by the employees.

#### Activity group 3 (ag3) - level and extent of mental task

Three of the four studies in *(ag3) - level and extent of mental task* examined associations with work factors [58, 65, 111]. They observed that tasks with higher mental demands are – to varying degrees – accompanied by higher control (time- and content-related), various and more challenging subtasks, better planning possibilities, and more qualification requirements. Workload, recorded as quantitative overload and time pressure in this study, increased with more automated and restrictive tasks, similar to the reports from *(ag1)- extent of technology use.*

In sum, associations from *(ag3)* highlight that an increased use of technologies seems to increase the occurrence of workload and time pressure.

## Discussion

The aim of this systematic review was to assess working conditions related to digitally connected work and to provide an overview of associations with various health and well-being outcomes. We identified and analyzed 14 studies from 1981 to 2019, five of them from the GDR [29, 58, 65, 109, 111]. A total of 5235 employees from the production and service sectors were examined. Despite of our focus on digital technologies in the search string, most included studies were conducted before 2000. The large time span of the studies, which thematically covers the introduction of automation and computerization to extensive digitization, is reflected in a heterogeneous study situation. The majority of the studies rely on “classical” working conditions (e.g., decision latitude, task variability) and established concepts and theories (e.g., Job-Demand-Control), regardless of the degree of digitization.

A major focus of all studies is on the work factor cluster *cognitive demands*. Especially its numerous combinations with the cluster *environmental factors/working tools* as well as the frequent connections to the cluster *organizational factors* are an indication of the growing complexity of work and working conditions with digitization mentioned in the introduction. *Social factors* on the other hand have hardly been researched. The identified relations to and effects on health and well-being resulting from these working conditions are diverse.

With regard to health outcomes, differences in the focus of old and new studies are striking. While older studies have a strong emphasis on strain-symptoms such as *physiological parameters and somatic complaints*, these do not play a role in the newer publications. The focus of the newer papers is clearly on psychological states like motivation and satisfaction as well as subjective evaluations (of affective symptoms). *Reduced well-being/affective symptoms* are researched in older and newer studies alike.

This difference might not primarily be attributable to digitization but rather to a general trend toward individualization within Western societies that is also reflected in the world of work. It puts the individual with their needs at the center of attention. The desire for appreciation of one’s own work performance or the demand for more individual flexibility are indicators of this subjectification of work [84]. However, in line with earlier research on the health impact of working conditions, a reversal of this trend in research can be observed: objective health outcomes gain new attention, and more and more research is being conducted in this field [74]. Modern sensor technology permits real-time monitoring of physiological parameters like heart rate and heart rate variability [93]. In addition, other rather new approaches such as immune markers are being pursued [75]. Using these developments in a multimethod approach including diverse dimensions of health could be helpful for a better understanding of relations and interactions of health and work factors in complex work systems.

Regarding work factors, the main cluster of *cognitive demands* includes important elements of a personality-promoting work design like “qualification”. This work factor was considered in old and new studies leading to the conclusion that fundamental skills are needed throughout the entire digitization process [4, 17, 29, 58, 65, 70, 114]. In addition to specific knowledge on the digital tools used, cognitive skills such as literacy, numeracy, and problem-solving are necessary as superordinate competencies [56].

Furthermore, the shift in the predominance of mental over physical tasks such as monitoring and controlling highlights a widely discussed digital dilemma: The possibility of simplifying or even replacing work processes results in deskilling for some employees, but at the same time also enforces a specialization of others. The conclusion that regular training as well as continuous education and participation seem to be required to meet the changing demands is not a new one [17, 19, 70]. But it does suggest that the possibility of continuous qualification as a part of personality-promoting work design will continue to be of importance in increasingly complex systems [100, 119].

Although individual qualification and competence are important, organizational factors are decisive for the effects of digitally connected work [88]. This is also reflected in relevant organizational work factors identified in the studies, above all workload and time pressure [4, 17, 29, 32, 58, 109, 114]. Furthermore, the influence of work organization and task design on the health of employees is often rated higher than or equally high to the influence of technologies per se: for example, Dainoff et al. [32] show that pressure is not dependent on the pure time spent at the computer. Rutenfranz et al. [109] conclude that the design of breaks has a greater influence on the health of employees than the machines they work on, and Umans et al. [117] show a moderating effect of corporate culture on the relation between the design of the digital work environment and job satisfaction. Concluding, Hacker and Schönfelder [58] see the critical influence on employees’ health and wellbeing in the organization of work and thus do not expect a “compelling relationship” between the introduction of computers and the improvement of working conditions, and Jackisch et al. [65] conclude that health consequences are generally caused by a complex set of conditions rather than isolated cause-effect relationships. Taken together, technology applications are always embedded in the design and organization of work which, in turn, should be included in analyses [70]. The complex set of conditions resulting from the embedding of technological tools seems decisive for the effects of digitally connected work [85, 88] and an isolated evaluation of tools and systems without considering the organizational context is not recommendable [23, 34].

In line with this reasoning, it seems important that work design reflects technological developments to evolve concurrently. Interestingly, older studies in this review, contrary to newer ones, examined technologies not only in terms of varying usages, but often with a deep reflection of their function [29, 32, 58, 65, 99, 109, 114]. This has been dispensed in the newer studies which is certainly due to the fact that the use of technological tools and applications is omnipresent nowadays [22, 41]. Additionally, the ergonomic aspects in work design were only approached in older texts [32, 114], probably due to emerging ergonomic standards such as DIN EN ISO 6385 [39] and the implementation of further occupational health and safety rules over time. But it is particularly striking that the work-factor cluster *environmental factors/working tools*, compared to others, has been most often considered in connection with other work factors like work organization, such as the association of interruptions due to technical reasons with increased time pressure [58, 70, 109]. Assuming that organizational structures and technology are fusing more and more with increasing connectivity/complexity of work, the reflection of technological functions and their embeddedness in work organization might be of increasing importance for the advancement of work design in digitally connected work.

One limitation of this systematic review could be the search string. It was not restricted to a particular time in order to identify possible transitions. We assumed that a search string that reflects newest digital technologies would set an automatic limit and clear focus. Nevertheless, we found a high degree of studies rooted in early days of digitally connected work. A possible explanation for this might be that we included new technologies in the search string, but also terms of human-computer interaction. Some of them might have already been used in the early days of computerization but are – with a different connotation – still relevant in digital connected work systems today. At the same time this enables an essential result of our review. We revealed many similarities concerning terms, working conditions and work design research between the first wave of automation and current digitalization. This points more to a gentler digital transition than to the often stated “disruptive technological change” [28]. A similar conclusion is drawn by Diebig et al. [36] concerning the relevance of classical working conditions in industry 4.0.

Nevertheless, the covered time span was a challenge for the assessment of the study quality and the summary of the results. The used QATSDD tool is well suited for the combination of quantitative, qualitative, and mixed-method studies, but the evaluation of older texts (especially from the GDR) was difficult with this approach. The quality of the studies cannot necessarily be judged worse by a lower overall score since many of the standards in this tool have been established only in recent years. An individual consideration of the respective results is therefore important for an overall assessment of the papers.

Based on the described heterogeneous study selection and the associated different approaches, it is not possible to carry out statistical evaluations or even a meta-analysis. A comparatively uniform approach for the evaluation and analysis of the results across studies was achieved by clustering the working conditions according to the risk assessment criteria of the European Commission [42].

In conclusion, even as the digital tools themselves have changed in the development from computerization and automation to digitization, this systematic review shows that the prevailing working conditions, such as cognitive demands, time pressure or workload, and their underlying work design theories remain relevant over time, even though their relations and importance shifted. Unfortunately, not all of the identified health outcomes and/or working conditions were analyzed in relation to each other. Our form of clustering reveals such gaps in research. Nevertheless, the associations found between the different clusters show that many factors can act as complexity drivers in a technologized work environment that might impact strain of employees in a multitude of ways. Future research should combine the context-rich approach of older studies with new methodological developments to cover this complexity and advance health- and personality-promoting work design in digitally connected work.

## Data Availability

availability of data is not applicable. The materials used are available from the corresponding author.

## References

[1] Alioth A, Udris I (1977). Fragebogen zur subjektiven Arbeitsanalyse (SAA).

[2] Allen TD, Johnson RC, Kiburz KM, Shockley KM (2013). Work–family conflict and flexible work arrangements: deconstructing flexibility. Pers Psychol.

[3] Andrews FM, Withey SB (1974). Developing measures of perceived life quality: results from several national surveys. Soc Indic Res.

[4] Andries F, Bijleveld CJH, Pot FD (1991). Working conditions and mental strain of automation personnel. Int J Hum Comput Interact.

[5] Armstrong-Stassen M (2001). Reactions of older employees to organizational downsizing the role of gender, job level, and time. J Gerontol B Psychol Sci Soc Sci.

[6] Arntz M, Gregory T, Zierahn U: *The risk of automation for jobs in OECD countries: A comparative analysis*. OECD Social, Employment and Migration Working Papers Nr. 189. Paris; 2016.

[7] Axtell C, Wall T, Stride C, Pepper K, Clegg C, Gardner P, Bolden R (2002). Familiarity breeds content: the impact of exposure to change on employee openness and well-being. J Occup Organ Psychol.

[8] Axtell CM, Fleck SJ, Turner N (2004). Virtual teams: collaborating across distance. Int Rev indus and organ Psychol.

[9] Ayyagari R, Grover V, Purvis R (2011). Technostress: technological antecedents and implications. MIS Q.

[10] Baethge A, Rigotti T (2013). Interruptions to workflow: their relationship with irritation and satisfaction with performance, and the mediating roles of time pressure and mental demands. Work & Stress.

[11] Bakker AB, Demerouti E (2007). The job demands-resources model: state of the art. J Manag Psychol.

[12] Barber LK, Santuzzi AM (2015). Please respond ASAP: workplace telepressure and employee recovery. J Occup Health Psychol.

[13] Berg-Beckhoff G, Nielsen G, Ladekjær Larsen E (2017). Use of information communication technology and stress, burnout, and mental health in older, middle-aged, and younger workers - results from a systematic review. Int J Occup Environ Health.

[14] Boswell WR, Olson-Buchanan JB (2007). The use of communication technologies after hours: the role of work attitudes and work-life conflict. J Manag.

[15] Bouchard TJ: Field research methods: interviewing questionnaires, participant observation, systematic observations, unobstrusive measures**.** *In Handbook of industrial and organizational psychology* edited by Dunnette MD. Chicago; 1976.

[16] Bradburn N. The structure of psychological well-being. Chicago; 1969.

[17] Brenner SO, Östberg O: Working conditions and environment after a participative automation project**.** Int J Ind Ergon 1995, 15:379–387. doi: 10.1016/0169-8141(94)00084-G, 5.

[18] Brod C (1982). Managing technostress: optimizing the use of computer technology. Pers J.

[19] Brougham D, Haar J (2018). Smart technology, artificial intelligence, robotics, and algorithms (STARA): employees’ perceptions of our future workplace. J Manag Organ.

[20] Brown R, Duck J, Jimmieson N (2014). E-mail in the workplace: the role of stress appraisals and normative response pressure in the relationship between e-mail stressors and employee strain. Int J Stress Manag.

[21] Brynjolfsson E, McAfee D. The second machine age: work, Progress, and prosperity in a time of brilliant technologies. New York; 2014.

[22] Carayon P: Healthy and efficient work with computers and information and communications technology—are there limits? SJWEH 2007, (Suppl 3):10–16.

[23] Carayon P, Smith MJ: Work organization and ergonomics**.** Appl Ergon 2000, 31:649–662. 10.1016/S0003-6870(00)00040-5, 6. 10.1016/s0003-6870(00)00040-5 11132049

[24] Cascio WF, Montealegre R (2016). How technology is changing work and organizations. Annu Rev Organ Psych Organ Behav.

[25] Cavanaugh MA, Boswell WR, Roehling MV,Boudreau JW: **An empirical examination of self-reported work stress among U.S. managers.** J. Appl. Psycho. 2000, 85:65–74. 10.1037/0021-9010.85.1.65 10740957

[26] Chatman JA, Spataro SE (2005). Using self-categorization theory to understand relational demography-based variations in people’s responsiveness to organizational culture. Acad Manag J.

[27] Chesley N (2014). Information and communication technology use, work intensification and employee strain and distress. Work Employ Soc.

[28] Christensen CM. The Innovator's dilemma: when new technologies cause great firms to fail. Boston; 1997.

[29] Claussner C, Müller W (1989). Arbeitsgestaltung und Arbeitserleben in der flexiblen automatisierten Fertigung - Analyse eines Beispiels aus dem VEB Kombinat Carl Zeiss JENA. Z Hyg.

[30] Corbett JM (1987). A psychological study of advanced manufacturing technology: the concept of coupling. Behav Inform Tech.

[31] Dainoff MJ, Hurrell JJ, Happ A: **A taxonomic framework for the description and evaluation of paced work.** *In Machine pacing occupational stress* edited by Salvendy G, Smith MJ. London; 1981:185–190.

[32] Dainoff MJ, Happ A, Crane P (1981). Visual Fatigue in VDT Operators. Hum Factors.

[33] Davenport TH, Kirby J: **Just how smart are smart machines?** MIT Sloan Manag. Review 2016, 57:21–25. http://sloanreview.mit.edu/article/just-how-smart-are-smart-machines.

[34] Day A, Paquet S, Scott N, Hambley L: Perceived information and communication technology (ICT) demands on employee outcomes: the moderating effect of organizational ICT support**.** J Occup Health Psychol 2012, 17(4):473–491. https://psycnet.apa.org/doi/10.1037/a0029837. 10.1037/a0029837 23066697

[35] Derks D, Bakker AB (2014). Smartphone use, work-home interference, and burnout: a diary study on the role of recovery. Appl Psychol: An Intern Review.

[36] Diebig M, Müller A, Angerer P (2017). Psychische Belastungen in der Industrie 4.0. Eine selektive Literaturübersicht zu (neuartigen) Belastungsbereichen. ASU Arbeitsmed Sozialmed Umweltmed.

[37] Diener ED, Emmons RA, Larsen RJ, Griffin S (1985). The satisfaction with life scale. J Pers Assess.

[38] Dijkstra A, van der Grinten MP, Schlatmann MJT, de Winter CR (1986). Functioning in the work situation.

[39] DIN EN ISO 6385: **Grundsätze der Ergonomie für die Gestaltung von Arbeitssystemen.** Berlin; 2016.

[40] Dragano N, Lunau T (2020). Technostress at work and mental health: concepts and research results. Curr Opin Psychiatry.

[41] Duradoni M, Innocenti F, Guazzini A: **Well-Being and Social Media: A Systematic Review of Bergen Addiction Scales.** Future Internet 2020, 12 (24). 10.3390/fi12020024.

[42] European Commission (1996). Guidance on risk assessment at work.

[43] Eyrolle H, Cellier JM (2000). The effects of interruptions in work activity: field and laboratory results. Appl Ergon.

[44] Fischer T, Riedl R (2017). Technostress research: a nurturing ground for measurement pluralism?. Commun Assoc Inf Syst.

[45] Franke F (2015). Is work intensification extra stress?. J Pers Psych.

[46] Frankenhaeuser M: **Stress at work. Threat, challenge, opportunity**. *In Dignity at work* edited by Stockholm E. 1985:128–139.

[47] Frankenhaeuser M, Johansson G (1986). Stress at work: psychobiological and psychosocial aspects. Appl Psychol.

[48] Fransella F, Bannister D. A manual for repertory grid technique. London; 1977.

[49] Ghislieri C, Molino M, Cortese CG (2018). Work and organizational psychology looks at the fourth industrial revolution: how to support workers and organizations?. Front Psychol.

[50] Glaser J, Seubert C, Hornung S, Herbig B (2015). The impact of learning demands, work-related resources, and job stressors on creative performance and health. J Pers Psychol.

[51] Goldberg DP. The detection of psychiatric illness by questionnaire. Oxford; 1972.

[52] Goldberg DP: Manual of the general health questionnaire*.* Windsor; 1978.

[53] Gottschall K, Voß G. Entgrenzung von Arbeit und Leben: zum Wandel der Beziehung von Erwerbstätigkeit und Privatsphäre im Alltag. München; 2000.

[54] Graf B, Antoni CH (2021). The relationship between information characteristics and information overload at the workplace - a meta-analysis. Europ J Work Organ Psychol.

[55] Greenhaus J, Parasuraman S, Wormley W (1990). Effects of race on organizational experiences, job performance evaluations, and career outcomes. Acad Manag J.

[56] Grundke R, Jamet S, Kalamova M, Keslair F, Squicciarini M (2017). Skills and global value chains: a characterization.

[57] Hacker W, Iwanowa A. Das Tätigkeitsbewertungssystem (TBS) – ein Hilfsmittel beim Erfassen potentiell gesundheits- und entwicklungsfördernder objektiver Tätigkeitsmerkmale. Psy Praxis. 1983;2:104–13.

[58] Hacker W, Schönfelder E (1985). Analyse und Bewertung der Arbeitsteilung und -kombination sowie der Mensch-Rechner-Funktionsteilung bei Arbeitstätigkeiten mit Bildschirmtechnik. Wiss Z Techn Univ Dresden.

[59] Hackman JR, Oldham GR: Development of the job diagnostic survey**.** J. Appl. Psychol. 1975, 60(2):159–170. https://psycnet.apa.org/doi/10.1037/h0076546.

[60] Harris K, Marett K, Harris R (2011). Technology-related pressure and work–family conflict: Main effects and an examination of moderating variables. J Appl Soc Psychol.

[61] Hickson D, Pugh D, Pheysey D (1969). Operations technology and organizational structure: an empirical reappraisal. Adm Sc Quarterly.

[62] Hoeck K, Hess H. Der Beschwerdefragebogen – BFB. Berlin; 1975.

[63] Humphrey SE, Nahrgang JD, Morgeson FP (2007). Integrating motivational, social, and contextual work design features: a meta- analytic summary and theoretical extension of the work design literature. J. Appl. Psychol..

[64] Iwanowa A, Hacker W: *Kurzverfahren zur Arbeitszufriedenheit.* (Forschungsbericht Bd. 46). TU Dresden, Institut für Allgemeine Psychologie und Methoden der Psychologie; 1997.

[65] Jackisch D, Richter PG (1989). Psychophysiologische Beanspruchungsuntersuchungen bei Bildschirmarbeit – zum Zusammenhang zwischen aktuellen und langfristigen Beanspruchungsfolgen, deren Ursache-Wirkungsbeziehung und Prozeßcharakter. Wissen Z Tech Univ Dresden.

[66] Karasek RA (1979). Job demands, job decision latitude and mental strain: implications for job redesign. Adm Sci Q.

[67] Kaufmann I, Pornschlegel H, Udris I. Arbeitsbelastung und Beanspruchung. In Belastungen und Stress bei der Arbeit edited by Zimmermann L, Reinbek. 1982:13–48.

[68] Kelloway E, Gottlieb B, Barham L (1999). The source, nature, and direction of work and family conflict: a longitudinal investigation. J Occup Health Psychol.

[69] Kopetz, H: *Simplicity is complex: foundations of cyber-physical system design*. Springer. 2019. DOI: https://doi-org.emedien.ub.uni-muenchen.de/10.1007/978-3-030-20411-2, 2019.

[70] Körner U, Müller-Thur K, Lunau T, Dragano N, Angerer P, Buchner A (2019). Perceived stress in human–machine interaction in modern manufacturing environments—results of a qualitative interview study. Stress Health.

[71] La Torre G, Esposito A, Sciarra I, Chiapetta M (2018). Definition, symptoms and risk of technostress: a systematic review. Int Arch Occup Environ Health.

[72] Lazarus RS. Psychological stress and the coping process. New York; 1966.

[73] Lazarus RS, De Longis A, Folkman S, Gruen R (1985). Stress and adaptional outcomes. The problem of confounded measures. Am Psychol..

[74] Lohani M, Payne BR, Strayer DL: **A Review of Psychophysiological Measures to Assess Cognitive States in Real-World Driving.** Front. Hum. Neurosci. 2019, 13(57). 10.3389/fnhum.2019.00057. 10.3389/fnhum.2019.00057 PMC6434408 30941023

[75] Marsland AL, Walsh C, Lockwood K, John-Henderson NA (2017). The effects of acute psychological stress on circulating and stimulated inflammatory markers: a systematic review and meta-analysis. Brain Behav Immun.

[76] Martin L, Omrani N (2015). An assessment of trends in technology use, innovative work practices and employees’ attitudes in Europe. Appl Econom.

[77] Maslach C, Jackson SE (1981). The measurement of experienced burnout. J Occ Behav.

[78] Matern B: *Spezielle Arbeits- und Ingenieurspsychologie.* Bd.3: Psychologische Arbeitsanalysen. Berlin; 1983.

[79] Mattioli S, Zanardi F, Baldasseroni A: Search strings for the study of putative occupational determinants of disease**.** Occup Environ Med 2010, 67:436–443. , 7, DOI: 10.1136/oem.2008.044727. 10.1136/oem.2008.044727 PMC2989170 19819858

[80] McNair DM, Lorr M, Droppelman LF: Manual: profile of mood states*.* San Diego; 1971.

[81] Merten F, Gloor P (2010). Too much e-mail decreases job satisfaction. Procedia – Soc. Behav Sci..

[82] Meyer JP, Allen NJ, Smith CA: Commitment to organizations and occupations: extension and test of a three-component conceptualization**.** J Appl Psychol 1993, 78 (4):538–551. https://psycnet.apa.org/doi/10.1037/0021-9010.78.4.538.

[83] Moher D, Liberati A, Tetzlaff J, Altman DG (2010). PRISMA group: preferred reporting items for systematic reviews and meta-analyses: the PRISMA statement. Int J Surg.

[84] Moldaschl M, Voß G. Subjektivierung von Arbeit. München; 2003.

[85] Montealgre R, Cascio W (2017). Technology-driven changes in work and employment. Commun ACM.

[86] Nehring R (1982). Beitrag zur Analyse und Bewertung von Arbeitstätigkeiten – Entwicklung eines Verfahrens zur subjektiven Tätigkeitsbewertung.

[87] Nitsch JR: **Die Eigenzustandsskala (EZ-Skala) – ein Verfahren zur hierarchisch mehrdimensionalen Befindlichkeitsskalierung.** *In Beanspruchung im Sport* edited by Nitsch JR, Udris, I. Bad Homburg; 1976.

[88] Oborski P (2004). Man-machine interactions in advanced manufacturing systems. Int J Adv Manuf Technol.

[89] OECD: ***OECD Employment Outlook*** 2017, OECD publishing, Paris, 10.1787/empl_outlook-2017-en.

[90] Oztemel E, Gursev S (2020). Literature review of Industry 4.0 and related technologies. J. Intell. Manuf.

[91] Palm E, Glaser J, Heiden B, Herbig B, Kolb S, Nowak D, Herr C (2016). Zusammenspiel von organisationalen Normen, individuellen Präferenzen und arbeitsbezogenem Entgrenzungsverhalten mit Konflikten zwischen Arbeits- und Privatleben. Wirtschaftspsychologie.

[92] Paulsson K, Sundin L (2000). Learning at work - a combination of experience based learning and theoretical education. Behav Info Technol.

[93] Peake JM, Kerr G, Sullivan JP. A critical review of consumer wearables, mobile applications, and equipment for providing biofeedback, monitoring stress, and sleep in physically active populations. Front Physiol. 2018;9. 10.3389/fphys.2018.00743. 10.3389/fphys.2018.00743 PMC6031746 30002629

[94] Petropoulos G: **The Impact of Artificial Intelligence on Employment.** In *The Fourth Industrial Revolution: Opportunities and Threats Work and Welfare* edited by O'Reilly J, Neufeind M, Ranft F.. London; 2018:119–132.

[95] Plath HE, Richter P (1984). Ermüdung – Monotonie – Sättigung – Stress.

[96] Plath HE, Richter P. Der BMS-II-Erfassungsbogen. Psychol Praxis Suppl. 1986:64–70.

[97] Ragu-Nathan TS, Tarafdar M, Ragu-Nathan BS, Tu Q (2008). The consequences of technostress for end users in organizations: conceptual development and empirical validation. Inf Sys Research.

[98] Rau R: Handlungssicherheit bei der Dispatchertätigkeit im Elektroenergieverorgungssystem – eine psychophysiologische Untersuchung*.* Frankfurt/M.; 1994.

[99] Rau R (1996). Einzelfallanalysen zur Bewertung von Handlungssicherheit in komplexen, automatisierten Systemen. Z Arbeits- und Organisationspsychol.

[100] Rau R (2006). Learning opportunities at work as predictor for recovery and health. Europ J Work Orga Psychol.

[101] Redden ES, Elliott LR, Barnes MJ: **Robots: the new teammates.** In *The Psychology of Workplace Technology* edited by Coovert MD, Thompson LF. N Y; 2014:185–208.

[102] Reeves M, Levin S, Fink T, Levina A: Taming complexity. Harv Bus Rev 2020, January–February:113–119.

[103] Remes J, Mischke J, Krishnan M (2018). Solving the productivity puzzle: the role of demand and the promise of digitization. International Productivity Monitor, Centre for the Study of Living Standards.

[104] Richardson KM, Thompson CA (2012). High tech tethers and work-family conflict: a conservation of resources approach. Engineering Manag Research.

[105] Richter PG, Leuteritz PM, Glatz B (1983). Langzeiterfassungsbogen (LEB) – Anleitung zu einem strukturierten Interview.

[106] Riedl R, Kindermann J, Auinger A, Javor A (2012). Technostress from a neurobiological perspective. System breakdown increases the stress hormone cortisol in computer users. Business Inf Sys Engineering.

[107] Rohmert W, Landau K: Das arbeitswissenschaftliche Erhebungsverfahren zur Tätigkeitsanalyse (AET)*.* Bern; 1979.

[108] Rudolph E, Schönfelder E, Hacker W. Tätigkeitsbewertungssytem für geistige Arbeit mit/ohne Rechnerunterstützung (TBS-GA). Berlin; 1983.

[109] Rutenfranz J, Kylian H, Schmidt KH, Klimmer F, Bubser R, Brandenburg U (1989). Belastungs- und Beanspruchungsanalysen bei Überwachungstätigkeiten in einer vollautomatisierten Fertigung. Z Hyg.

[110] Salazar-Concha C, Ficapal-Cusi P, Boada-Grau J, Camacho L. Analyzing the evolution of technostress: a science mapping approach. Heliyon. 2021;7(4). 10.1016/j.heliyon.2021.e06726. 10.1016/j.heliyon.2021.e06726 PMC8065288 33912710

[111] Seibt A, Friedrichsen G, Geist HW, Schurig HU, Roehner J (1988). Untersuchungen zur Beanspruchung durch Schichtarbeit unter den Bedingungen automatisierter Fertigung. Z Hyg.

[112] Sirriyeh R, Lawton R, Gardner P (2012). Reviewing studies with diverse designs: the development and evaluation of a new tool. J Eval Clin Pract.

[113] Soucek R, Moser K (2010). Coping with information overload in email communication: evaluation of a training intervention. Comput Hum Behav.

[114] Stellman JM, Klitzman S, Gordon GC, Snow BR: Work environment and the well-being of clerical and VDT workers**.** J Organ Behav 1987, 8(2):95–114. http://www.jstor.org/stable/3000364, DOI: 10.1002/job.4030080202.

[115] Tarafdar M, Tu Q, Ragu-Nathan BS, Ragu-Nathan TS (2007). The impact of Technostress on role stress and productivity. J Manag Inf Sys.

[116] Tarafdar M, Cooper C, Stich JF (2019). The technostress trifecta - techno eustress, techno distress and design: theoretical directions and an agenda for research. Inf Sys J.

[117] Umans T, Kockum M, Nilsson E, Lindberg S (2018). Digitalisation in the banking industry and workers subjective well-being: contingency perspective. Int J Workplace Health Manag.

[118] Van de Ven A, Delbecq A (1974). A task contingent model of work unit structure. Admin Sc Quarterly.

[119] Van Ruysseveldt J, Verboon P, Smulders P: Job resources and emotional exhaustion. The mediating role of learning opportunities**.** Work & Stress 2011, 25(3):205–223. https://psycnet.apa.org/doi/10.1080/02678373.2011.613223.

[120] Warr P. Work environment and mental health. New York; 1987.

[121] Warr PB, Cool JD, Wall TD (1979). Scales for the measurement of some work attitudes and aspects of psychological well-being. J Occup Psychol.

[122] Zam – Zentralinstiut für Arbeitsmedizin der DDR (Eds.): *Arbeitsmedizinische Tauglichkeits- und Überwachungsuntersuchung. Teil 1 und 2.* Berlin; 1980.

[123] Zammuto RF, Griffith TL, Majchrzak A, Dougherty DJ, Faraj S (2007). Information technology and the changing fabric of organization. Organ Sci.

[124] Zuboff S. In the age of the smart machine: the future of work and power. New York; 1988.

